# Clinical Effectiveness of Platelet-Rich Plasma for Long-Bone Delayed Union and Nonunion: A Systematic Review and Meta-Analysis

**DOI:** 10.3389/fmed.2021.771252

**Published:** 2022-01-25

**Authors:** Shang Li, Fei Xing, Rong Luo, Ming Liu

**Affiliations:** ^1^Department of Plastic and Burn Surgery, West China Hospital, Sichuan University, Chengdu, China; ^2^Department of Orthopaedics, West China Hospital, Sichuan University, Chengdu, China

**Keywords:** platelet-rich plasma, delayed union, nonunion, systematic review, long-bone

## Abstract

**Background:**

More recently, there was a series of clinical studies focusing on local administration of platelet-rich plasma (PRP) in long-bone fracture patients suffering from delayed union and nonunion. Therefore, we performed a systematic review to evaluate the effectiveness of PRP injection for treatment of patients with long-bone delayed union and nonunion.

**Methods:**

Relevant clinical trials were selected by the main bibliographic databases, including Medline, PubMed, Embase, Web of Science, and the Cochrane library, to evaluate the effectiveness of PRP for long-bone fracture patients diagnosed with delayed union or nonunion by two reviewers independently. The main outcomes included healing rate, healing duration, pain relief, functional outcome, and complications.

**Results:**

Finally, thirteen studies including four hundred and fifty-nine participants met the selection criteria and were included in this systematic review. These articles included three randomized controlled studies, one prospective study, and nine retrospective studies. 146 out of 155 (94.19%) patients treated with PRP during operation, and 144 out of 183 (78.69%) patients treated with PRP injection alone, exhibited bone consolidation. The healing rate of the PRP group (85.80%) was higher than that of the control group (60.76%). The mean bony union time of patients treated and untreated with PRP, were, respectively, 4.64 and 5.15 months. Four papers reported that PRP was effective in pain relief of patient with delayed union and nonunion. Complications, including small subcutaneous hematoma, subcutaneous swelling, and postoperative infection, were also reported in enrolled studies.

**Conclusions:**

PRP is a promising alternative treatment for patients with long-bone delayed union and nonunion. PRP could successfully promote the healing rate and relieve the pain of patients with delayed union and nonunion. However, the long-term adverse side and functional outcome in application of PRP still need further large-scale trials and long-term follow-up.

**Systematic Review Registration:**

https://www.researchregistry.com/browse-the-registry#home/registrationdetails/61dbd2f837e948001e68d5c5/, The identifying number is research registry 7525.

## Introduction

The bone tissue has the potential of spontaneous healing after injuries. However, the regenerative capacity of bone tissue is limited (1). In addition, large bone defects, multiple fractures, and bone metabolic diseases may result in long-bone delayed union or nonunion, which are the most devastating complications of traumatic fractures (2). Currently, the principle of treatment for long-bone delayed union or nonunion includes local infection control, debridement, deformity correction, fixation, bone graft, and biological stimulus (3). However, this treatment has the disadvantages of long treatment period, operation trauma to the patient, long time recovery, limited source of autologous bone, and high treatment cost. Due to its limitation, many alternative therapeutic methods, used either alone or in combination with surgery, have been implemented in recent years (4).

The growth factor is one of the major components in the complex bone healing process and could activate and regulate many aspects of osteoblasts, osteoclasts, and stem cells (5). Deriving from the multiple centrifugations of the own peripheral blood, platelet-rich plasma (PRP) includes considerable amounts of growth factors, such as transforming growth factor, platelet-derived growth factor, endothelial growth factor, vascular endothelial growth factor, fibroblast growth factor, insulin-like growth factor (6). Recently, numerous basic experimental studies have confirmed the potential therapeutic value of platelet-rich plasma (PRP) in repair of bone and cartilage tissues (7). Although PRP repair mechanism involved still remains unclear, the easy application procedure of PRP and its possible beneficial effects make PRP a promising therapeutic method for delayed union and nonunion (8).

More recently, there was a series of clinical or preclinical studies focusing on local administration of PRP in long-bone fracture patients suffering from delayed union and nonunion. However, the efficacy and safety of PRP for patients with long-bone delayed union and nonunion still remains controversial. As there was no related systematic review published yet, we performed the present systematic review to study the clinical efficacy and safety of PRP injection in patients with long-bone delayed union and nonunion.

## Methods

The Preferred Reporting Items for Systematic reviews and Meta-Analysis (PRISMA) guidelines were followed (9). In our study, we created a systematic review, consisting of objectives, study selection strategies, inclusion and exclusion criteria, statistical analysis, and outcome measures.

### Literature Search

Two reviewers independently searched for potentially relevant published and unpublished studies using electronic databases, including Cochrane Central Register of Controlled Trials (April 2020), MEDLINE, PubMed (1966 to April 2020), Web of Science (1990 to April 2020), and Embase (1974 to April 2020). We also used the Google search engine (April 2020) to search for additional eligible studies. The following keywords were used for the search: “platelet-rich plasma,” “PRP,” “autologous plasma,” “delayed union,” “nonunion,” “non-union,” “fracture delayed union,” “fracture nonunion.” This strategy was adapted for each included electronic database, and no specific database filters were applied. Human *in vivo* studies on the treatment of long-bone delayed union and nonunion with PRP were searched. We initially assessed the titles and abstracts of the search results, and then performed a careful review of the full-text. To maximize the search, we also used a method of backward chaining of references from retrieved papers.

### Eligibility Criteria

Inclusion criteria for this systematic review were as follows: (1) The studies include prospective randomized control studies, retrospective control studies, and retrospective cohort studies. (2) Studies are focusing on patients suffering from long-bone delayed union or nonunion, for which there have been no clinical and radiological signs of healing for 3 months or more. (3) The experimental group involves administration of PRP alone or PRP combined with surgery. While placebo or surgery alone is involved in control group. (4) Unpublished studies were also included. (5) Publishing language is English. (6) The studies report the procedures of PRP injection performed in participants.

The exclusion criteria for this study were as follows: (1) Case reports, comment papers, and correspondence. (2) Animal studies. (3) Protocol description. (4) Publishing language is not English. (5) Duplicated publications. If there is a dispute between the two reviewers, it will be settled through consultation or consultation with a third reviewer.

### Data Extraction

Data of patients with long-bone delayed union and nonunion in each study were independently extracted by two reviewers. The demographic characteristics extracted for systematic review were as follows: first author, year of publication, medical center, study format, sample size in each study, average age of patients, male ratio, follow-up. The interventional factors extracted for systematic review were as follows: types of nonunion, types of long-bone, surgical intervention, PRP dosage, controls. All data were entered into an electronic spreadsheet. Furthermore, any disagreements were resolved by discussion and consensus with a third reviewer. Descriptive statistics were calculated for each study and parameters were analyzed.

### Outcome Measures

The clinical outcomes of this systematic review comprise primary outcomes and secondary outcomes, the primary outcomes including healing rate and healing duration, the secondary outcomes including pain relief, functional outcome, and complications. We evaluated the clinical efficacy and safety of PRP injection in patients with long-bone delayed union and nonunion.

### Assessment of Methodological Quality

The methodological quality of randomized controlled studies in this study was independently evaluated by two reviewers. Modified Jadad scores were used to evaluate the bias of randomized comparative studies in this study (10). Studies were considered to be of high quality when modified Jadad scores ≥ 4 points from a possible total of eight. The Methodological Index for Non-Randomized Studies (MINORS) was applied to evaluated non-randomized comparative or non-comparative studies by two reviewers independently (11). The items included in MINORS for non-comparative studies were as follows: (1) A clearly stated aim (2) Inclusion of consecutive patients (3) Prospective collection of data (4) Endpoints appropriate to the aim of the study (5) Unbiased assessment of the study endpoint (6) Follow-up period appropriate to the aim of the study (7) Loss to follow up <5% (8) Prospective calculation of the study size. Additional criteria in the case of comparative study were as follows: (9) An adequate control group. (10) Contemporary groups (11) Baseline equivalence of groups (12) Adequate statistical analyses. The items are scored 0 (not reported), 1 (reported but inadequate) or 2 (reported and adequate). Furthermore, any disagreements were resolved by discussion and consensus with a third reviewer.

### Statistical Analysis

The statistical analysis was independently performed using RevMan Manager 5.3 (The Cochrane Collaboration, Oxford, UK) by two reviewers. *P* < 0.05 was considered statistically significant. For continuous variables, such as healing duration, weight mean differences (WMDs) were estimated with a 95% confidence interval (95% CI). For dichotomous data, such as healing rate, the odds ratio (OR) was estimated with a 95% confidence interval (95% CI). Statistical heterogeneity for all enrolled studies was evaluated using the Q chi-square test and I2 statistic.

## Results

### Study Selection

**Figure 1:**
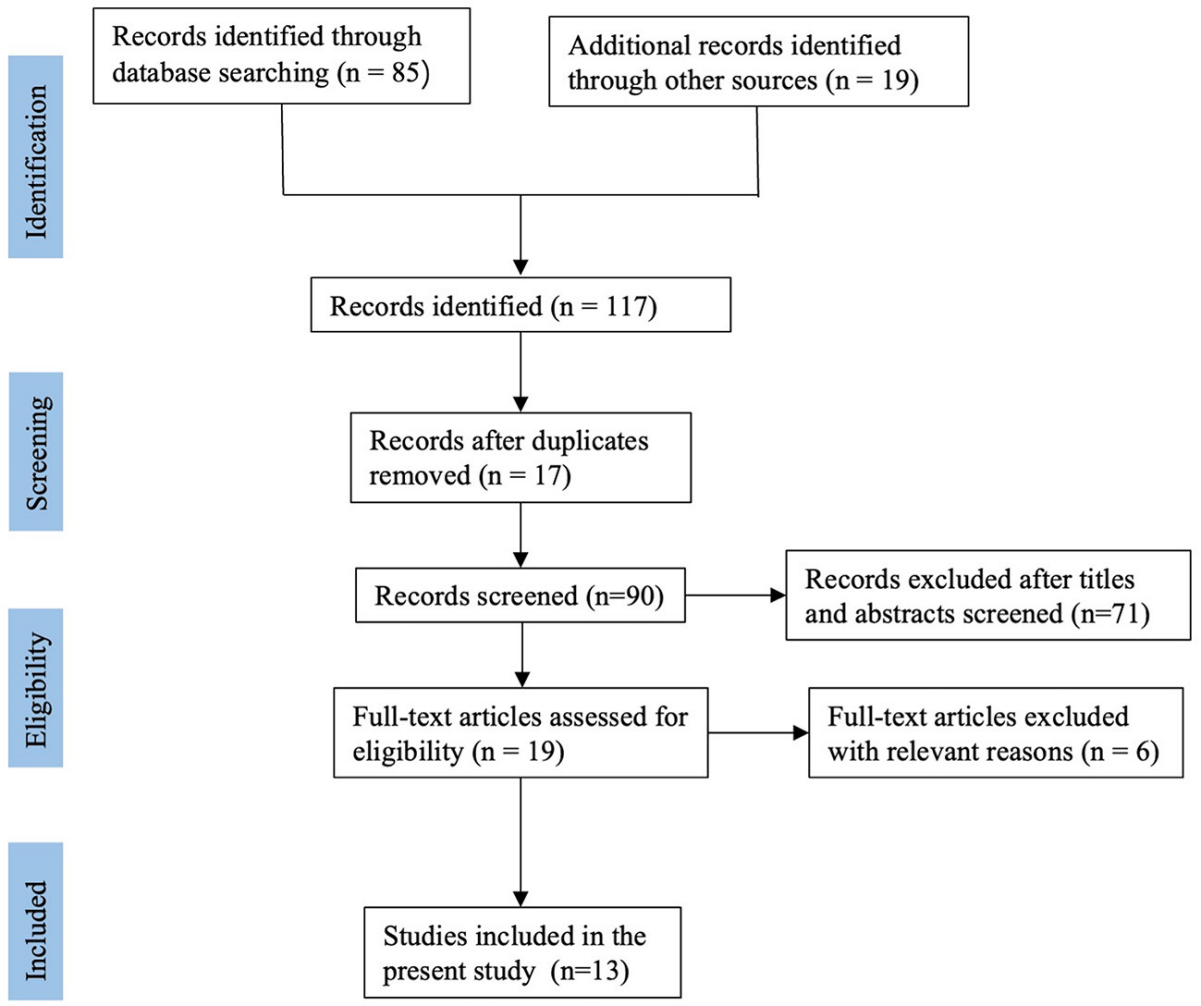
The flow diagram involved in the current study.

After literature search, a total of 117 relevant publications were retrieved. After excluded duplicate records, 90 studies were screened using their titles and abstracts. Finally, 13 studies including 459 participants met the selection criteria and were included in this systematic review. These articles include three randomized controlled studies (12–14), one prospective study (15), and nine retrospective studies (16–24). All enrolled studies were published between 2008 and 2020. The flow diagram involved in the current study is shown in Figure 1.

### Study Characteristics

Patients' characteristics are reported in Table 1. All 13 papers discussed the administration of PRP in treatment for long-bone delayed union and nonunion. A total of 459 patients were included in the present study (326 males, 130 females). The sample size of these studies ranged from 3 to 94. Among these included studies, five studies were conducted in Italy, two studies in Turkey, two studies in India, each one in Poland, Mexico, Venezuela and Iran. The mean age of the participants ranged from 14 to 60.6 years. In addition, the follow-up of enrolled studies ranged from 6 to 72 months.

**Table 1:** An overview of the included studies.

**References**	**Centre**	**Study format**	**Patients**	**Age (years)**	**Male**	**Follow up (months)**	**Outcome measures**
De Vitis et al. (24)	Italy	Consecutive cohort, retrospectively collected	45/42	31.79 ± 10.82	34/36	12	Healing duration, VAS, Mayo Wrist Score, quick-DASH
Duramaz et al. (22)	Istanbul, Turkey	Consecutive cohort, retrospectively collected	14/15	38.58 ± 10.1	8/8	24	Healing rate, VAS
Pozza et al. (21)	Turin, Italy	Consecutive cohort, retrospectively collected	25	40.4 ± 11.7	16	6	Healing rate
Samuel et al. (12)	Puducherry, India	Prospective randomized control trial	23/17	36.45(20-60)	23/14	9	Healing rate, healing duration
Campochiar et al. (20)	Modena, Italy	Consecutive cohort, retrospectively collected	9	60.6(44-70)	2	11-36	Healing rate, Pain, DASH, UCLA, bony union time
Carlos et al. (13)	Monterr, Mexico	Prospective randomized double-blind controlled trial	7/9	38.1 ± 12.98	5/8	9	Bone consolidation time, quick-DASH
Ghaffarpasand et al. (14)	Shiraz, Iran	Prospective randomized double-blind controlled trial	37/38	26.40 ± 6.01	31/33	9	Hospital stays, healing duration, pain
Wittig et al. (25)	Caracas, Venezuela	Consecutive cohort, retrospectively collected	3	50.33 ± 22.65	NR	14-36	Healing duration
Malhotra et al. (19)	New Delhi India	Consecutive cohort, retrospectively collected	94	NR	66	4	Healing rate
Memeo et al. (18)	Milan, Italy	Consecutive cohort, retrospectively collected	7	14(11-19)	6	9-72	Healing rate, bony union time
Say et al. (15)	Samsun, Turkey	Consecutive cohort, prospective collected	20	33.5(15-77)	17	≥8	Healing rate
Bielecki et al. (16)	Zabrze, Poland	Consecutive cohort, retrospectively collected	32	41.41(19-60)	25	10	Healing rate, hospital stay, BMD
Galasso et al. (17)	Catanzaro, Italy	Consecutive cohort, retrospectively collected	22	39(20-56)	13	13	Healing rate, bony union time

*VAS, Visual Analogue Scale; DASH, disabilities of the arm, shoulder, and hand; UCLA, the university of California at Los Angeles shoulder rating scale; NR, not reported*.

The intervention measures in patients with delayed union or nonunion of all enrolled studied were shown in Table 2. The type of long bone in enrolled studies included femur, tibia, fibula, humerus, ulna, radius, and clavicle. The type of nonunion included hypertrophic, oligotrophic, and atrophic nonunion. The PRP dosage ranged from 2.5 to 20 ml. Eight studies combined intraoperative administration of PRP with external or internal fixation surgeries in treatment for nonunion. Five studies used PRP alone in treatment.

**Table 2:** The intervention measures in patients with delayed union or nonunion of all enrolled studied.

**References**	**Types of nonunion**	**Types of long bone**	**Surgical intervention**	**PRP dosage**	**Control group**
De Vitis et al. (24)	Nonunion	Scaphoid	Osteosynthesis with shape memory staple and PRP	NR	No used PRP
Duramaz et al. (22)	Nonunion (oligotrophic)	Femur, tibia	-	10 ml	Changed nail
Pozza et al. (21)	Nonunion	Humerus, femurs, tibia	External, internal, or both fixation surgery, MSC and PRP performed in all	5 ml	-
Samuel et al. (12)	Delayed union	Tibia, femur, forearm, humerus	-	10 ml	No used PRP
Campochiar et al. (20)	Nonunion (atrophic)	Humerus	Angular stability plate associated, tibial cortical graft and allograft and PRP	NR	-
Carlos et al. (13)	Nonunion	Humerus	ORIF and iliac crest autograft	12 ml	No used PRP
Ghaffarpasand et al. (14)	Nonunion (hypertrophic, oligotrophic, atrophic)	Femur, tibia, humerus, ulna	Intramedullary nailing or ORIF, iliac crest autograft performed in all.	5 ml	5ml normal saline
Wittig et al. (25)	Nonunion	Tubia, fibula, femur	External or internal fixation surgery, and MSC/collagen microspheres/PRP performed in all	NR	-
Malhotra et al. (19)	Nonunion	Tibia, femur, humerus, radius, ulna	-	15–20 ml	-
Memeo et al. (18)	Nonunion	Ulna, radius	Unreamed locked intramedullary nail and PRP	10 ml	-
Say et al. (15)	Delayed union, nonunion	Femur, tibia	-	2.5 ml	-
Bielecki et al. (16)	Delayed union, nonunion	Tibia, femur, fibula, Humerus, radius, clavicle	-	3 ml	-
Galasso et al. (17)	Nonunion	Tibia, femur, humerus	Removal of previous implants, non-union ends decorticating, canal reaming, intramedullary nailing, PRP injection	6 ml	-

*NR, not reported*.

### Risk of Bias

**Figure 2:**
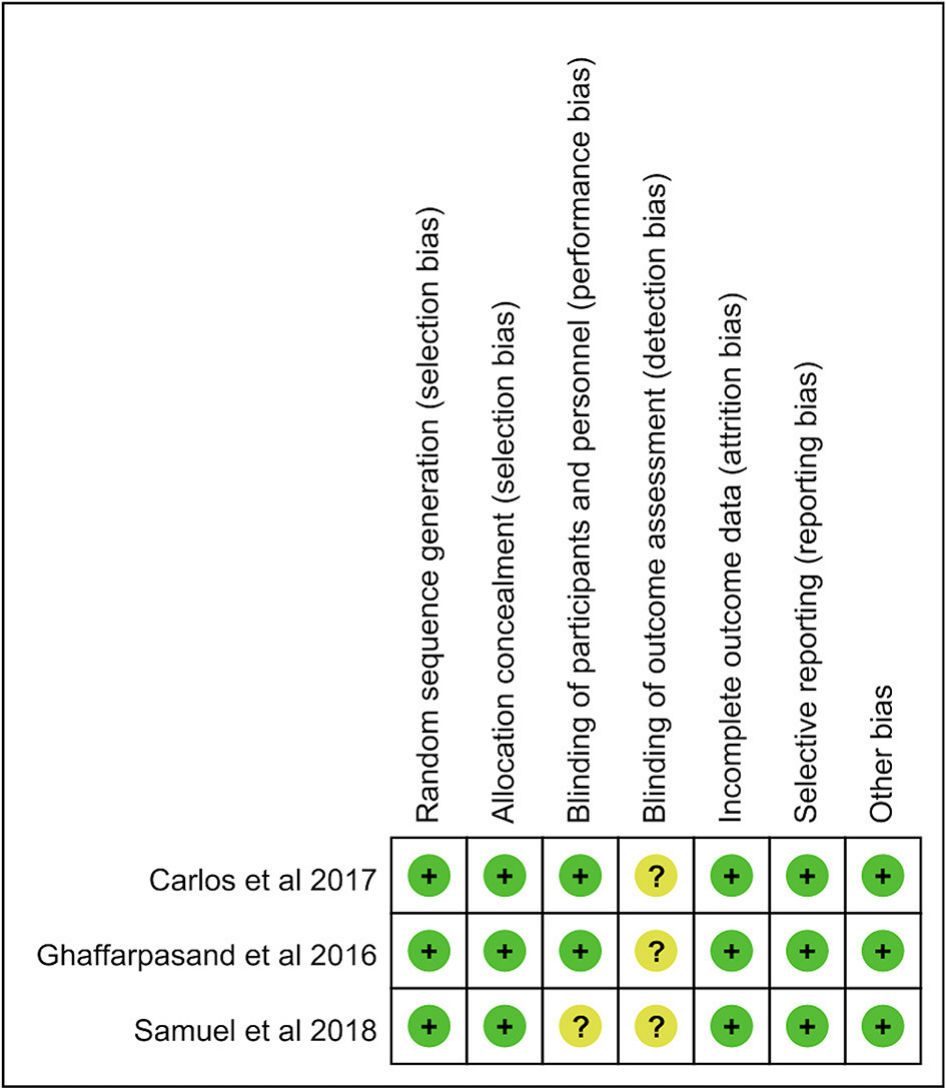
The risk of bias of all prospective clinical studies.

The risk of bias of randomized controlled studies in this study was independently evaluated by two reviewers. The modified Jadad scores of randomized controlled studies were shown in Table 3. The median of the modified Jadad score of the randomized controlled studies was 6 (range, 4–7), which indicated that all enrolled randomized controlled studies were considered to be of high quality. Additionally, risk of bias of all prospective clinical studies are shown in Figure 2. The MINORS scale was used to evaluate the methodological quality of non-randomized comparative or non-comparative studies in this study. The median of MINORS of the non-randomized studies was 13 (range, 13–22). The MINORS of non-randomized studies in this study was shown in Table 4. Eleven studies with a total of 108 items of risk of bias were assessed.

**Table 3:** Modified Jadad Score for clinical trials.

**Study (Year)**	**Randomization**	**Concealment of allocation**	**Double blinding**	**Total withdrawals and dropouts**	**Total**
Samuel et al. (12)	**		*	*	4
Carlos et al. (13)	**	**	*	*	6
Ghaffarpasand et al. (14)	**	**	*	**	7

*Each asterisk represents one point. Modified Jadad score is used to evaluate the quality of articles and studies achieving a score of ≥4 points were considered to be of high quality*.

**Table 4:** Summary of the risk of bias assessment for nonrandomized studies with the MINORS (Methodological Index for Non-Randomized Studies) criteria.

**References**	**A**	**B**	**C**	**D**	**E**	**F**	**G**	**H**	**I**	**J**	**K**	**L**	**Total**
De Vitis et al. (24)	2	2	2	2	0	2	2	0	2	2	2	2	20
Duramaz et al. (22)	2	2	1	2	2	2	2	0	2	2	2	2	21
Pozza et al. (21)	2	2	1	2	2	2	2	0	-	-	-	-	13
Campochiaro et al. (20)	2	2	1	2	2	2	2	0	-	-	-	-	13
Wittig et al. (25)	2	2	2	1	0	2	2	0	-	-	-	-	11
Malhotra et al. (19)	2	2	1	2	2	2	2	0	-	-	-	-	13
Memeo et al. (18)	2	2	1	2	2	2	2	0	-	-	-	-	13
Say et al. (15)	2	2	2	2	2	2	2	0	2	2	2	2	22
Bielecki et al. (16)	2	2	1	2	2	2	1	0	2	1	2	2	19
Galasso et al. (17)	2	2	1	2	2	2	2	0	-	-	-	-	13

*(A) A clearly stated aim. (B) Inclusion of consecutive patients. (C) Prospective collection of data. (D) Endpoints appropriate to the aim of the study. (E) Unbiased assessment of the study endpoint. (F) Follow-up period appropriate to the aim of the study. (G) Loss to follow up less than 5%. (H) Prospective calculation of the study size. (I). An adequate control group. (J) Contemporary groups. (K) Baseline equivalence of groups. (L) Adequate statistical analyses. The items are scored 0 (not reported), 1 (reported but inadequate) or 2 (reported and adequate)*.

### Primary Outcome

#### Healing Rate

All enrolled studies reported the healing rate of patients with delayed union or nonunion after treatment with PRP. Finally, 290 out of 338 (85.80%) patients treated with PRP, alone or in combination, exhibited bone consolidation. While the healing rate of the control group was 60.27%, these patients were treated by fixation surgery alone. 146 out of 155 (94.19%) patients treated with PRP during operation, exhibited bone consolidation. 144 out of 183 (78.69%) patients treated with PRP injection alone, exhibited bone consolidation. Healing rates of all groups in enrolled studies were shown in Table 5. Healing rates among groups of intraoperative administration of PRP with external or internal surgeries ranged from 81.1 to 100%. Healing rates among groups of PRP injection alone, ranged from 30 to 92.86%.

**Table 5:** Healing rates and healing duration of enrolled studies.

**References**	**Bony union time (months)**	**Healing rate**
De Vitis et al. (24)	PRP group: 2.1 ± 0.5; Control group: 2.9 ± 0.9	PRP group:100%; Control group:95.2%
Duramaz et al. (22)	PRP group: 4.18 ± 0.6; Control group: 4.7 ± 0.92	PRP group:92.86%; Control group:80%
Pozza et al. (21)	NR	100%
Samuel et al. (12)	PRP group: 3.83 ± 2.48; Control group: 3.28 ± 1.80	PRP group:78.26%; Control group:41.18%
Campochiar et al. (20)	7 (5-9)	100%
Carlos et al. (13)	PRP group: 4.98 ± 0.56; Control group: 6.36 ± 0.52	PRP group:100%; Control group:88.89%
Ghaffarpasand et al. (14)	PRP group: 8.1 ± 1.2; Placebo group: 8.5 ± 0.7	PRP group:81.1%; Placebo group:55.3%
Wittig et al. (25)	NR	100%
Malhotra et al. (19)	NR	87.2%
Memeo et al. (18)	5.75 (4-9)	100%
Say et al. (15)	3.75 (2-6)	30%;
Bielecki et al. (16)	2.8 ± 0.69	78.13%
Galasso et al. (17)	5.38(3-6.5)	91%

*NR, not reported*.

**Figure 3:**
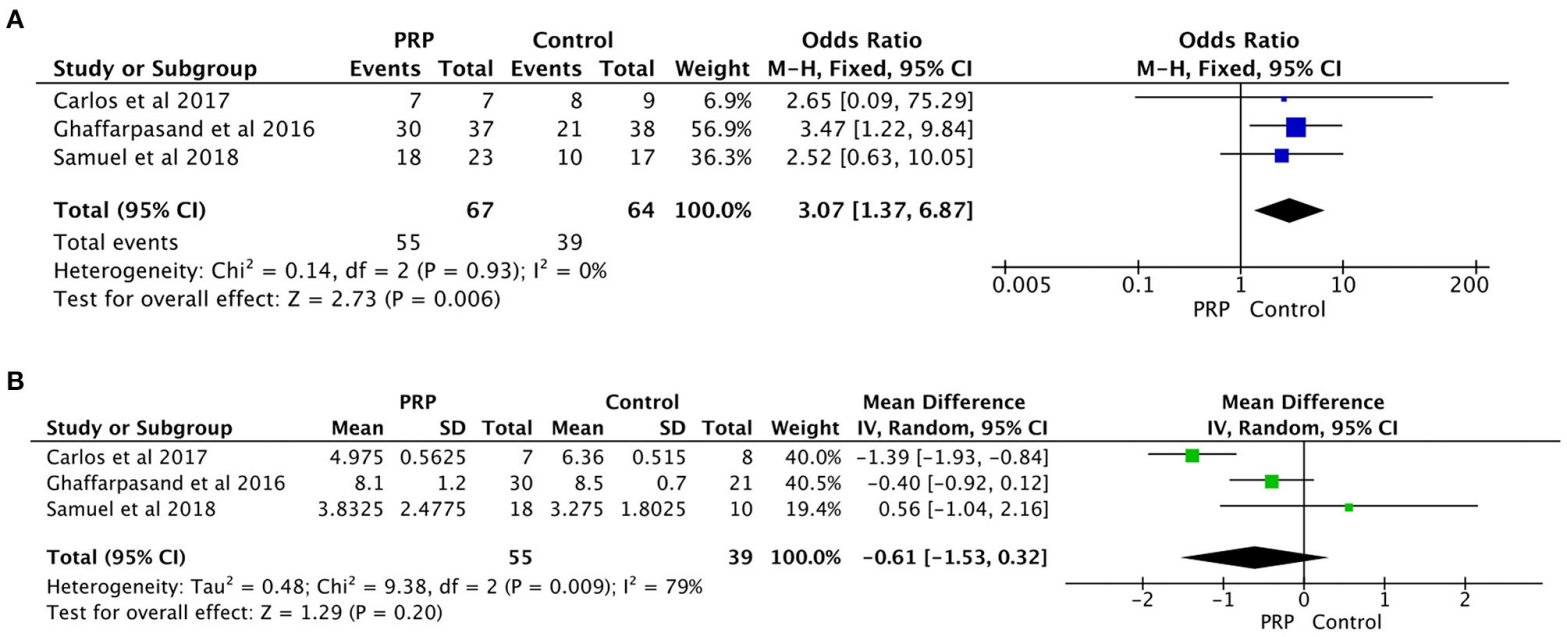
**(A)** The forest plot of healing rate. **(B)** The forest plot of healing duration.

Three prospective studies reported data on healing rate of both PRP and control group. The statistical results from these studies were enrolled in the meta-analysis (Figure 3A). The results showed that the use of PRP could increase the healing rate (OR = 3.07; 95%CI, 1.37-6.87; *P* = 0.006), and there was no significant heterogeneity in healing rate (*P* = 0.93, I^2^ = 0%).

#### Healing Duration

Ten out of thirteen studies reported the bony union time of patients, which was shown in Table 5. The mean bony union time in patients with delayed union or nonunion ranged from 2.8 months to 8.5 months. The mean healing duration of patients treated with PRP was 4.64 months. The mean healing duration of patients untreated with PRP was 5.15 months.

The statistical results from three studies were enrolled in the meta-analysis (Figure 3B). No significant differences were found between PRP group and control group in terms of healing duration (WMD = −0.61; 95%Cl: −1.53 to 0.32; *P* = 0.20). However, the results show high heterogeneity in healing duration (*P* = 0.009, I^2^ = 79%).

### Secondary Outcome

#### Pain Relief

Four out of thirteen studies, included 260 patients with long-bone nonunion, reported the pain relief effect of PRP injection (Table 6). Campochiar et al. (20) reported that the mean VAS scores decreased from 7 (5-9) preoperatively to 2 (0-3) at final follow-up, after receiving treatment of changing internal fixation, bone grafting, and PRP injection. Duramaz et al. (22) mentioned that injection of PRP alone or changing intramedullary nail could both significantly lower preoperative VAS scores of patients suffering from femur and tibia nonunion. In addition, the mean VAS values in preoperative and postoperative periods were similar in two groups of injection of PRP alone and changing intramedullary nail, respectively. Ghaffarpasand et al. (14) reported that the VAS scores of group combined surgery and PRP was significantly lower than that of surgery group at days 45, 90, and 135, postoperatively. However, there were no differences between two groups in days 180, 225, and 270, postoperatively. These trend were consistent with that reported by De Vitis et al. (24). They reported the VAS scores of groups combined surgery and PRP were significantly lower than that of surgery group at 3 months, postoperatively. While the VAS scores of two group tended to close after 6 months.

**Table 6:** Pain relief, functional outcome, and complications of enrolled studies.

**References**	**Pain relief**	**Functional outcome**	**Complications**
De Vitis et al. (24)	No difference in preoperative VAS, but 3 months postoperative VAS was different significantly of two groups. Postoperative VAS scores were significantly lower than that preoperative VAS scores, respectively	Quick-DASH score: Preoperative: PRP group (46.2 ± 6.2), Control group (47.7 ± 6.2); 3 months postoperative: PRP group (7.2 ± 4.6), Control group (12.6 ± 3.1)	NR
Duramaz et al. (22)	No difference in preoperative and postoperative VAS scores of two groups. Postoperative VAS scores were significantly lower than that preoperative VAS scores, respectively	NR	NR
Pozza et al. (21)	NR	NR	A small subcutaneous hematoma was detected in four patients
Samuel et al. (12)	NR	NR	NR
Campochiar et al. (20)	VAS score was 7 (5-9) preoperative and 2 (0-3) at final follow-up after healing (min 0–max 3)	Dash score:22.25(5.8-40.9), Constant score:64(45-79), UCLA score: 27(22-34)	NR
Carlos et al. (13)	NR	Quick-DASH score: PRP group (7.28 ± 11.30), Control group (5.32 ± 8.23)	NR
Ghaffarpasand et al. (14)	The VAS scores of PRP group was significantly lower than that of control group at days 45, 90, and 135.No differences in days 180, 225, and 270.	NR	Five patients in PRP group and two patients in control group associated with postoperative injection
Wittig et al. (25)	NR	NR	NR
Malhotra et al. (19)	NR	NR	NR
Memeo et al. (18)	NR	NR	NR
Say et al. (15)	NR	NR	NR
Bielecki et al. (16)	NR	NR	Subcutaneous swelling at the injection site
Galasso et al. (17)	NR	NR	NR

*NR, not reported*.

#### Function Outcome

Two of eleven studies reported the postoperative function outcome of humeral nonunion (Table 6). De Vitis et al. (24) reported the quick-DASH score of groups combined surgery and PRP significantly lower than that of surgery group at 3 months postoperatively. However, there was no difference between two groups after 6 months. Campochiar et al. (20) reported that after treatment with plate, cortical bone graft and PRP, the mean Dash, Constant, and UCLA scores were 22.25 (5.8-40.9), 64 (45-79), and 27 (22-34), respectively. Carlos et al. (13) reported that there were no significant differences between the iliac crest autograft group and the iliac crest autograft combined with PRP group at the end of follow-up.

#### Complications

Sixteen studies including 369 participants described whether complications occurred after administration of PRP in treatment for long-bone delayed union and nonunion (Table 6). Three kinds of complications within this study population occurred in three different studies. Ghaffarpasand et al. (14) reported that five patients in the PRP group were associated with the postoperative injection. Two of these five patients were treated with revision. The postoperative infection rate of the PRP group was higher than the control group, while this difference did not reach statistical significance. Pozza et al. (21) reported that a small subcutaneous hematoma was detected in four patients by contrast-enhanced ultrasound at seven days after the PRP injection. Subcutaneous hematoma resolved spontaneously at subsequent evaluations. Bielecki et al. (16) reported several patients developed subcutaneous swelling at the injection site, especially in treatment for tibial and fibular healing disturbances. Subcutaneous swelling at the injection site resolved spontaneously within several hours. In addition, several patients had moderate discomfort at their donor vein site, which generally resolved within several hours.

## Discussion

This systematic review aimed to evaluate the clinical efficacy and safety of administration of PRP in patients suffering from long-bone delayed union and nonunion. In addition, we found that PRP is a promising alternative treatment for delayed union and nonunion. All included studies in this systematic review were published from 2008 to 2020. Eight out of thirteen studies were published in the past 5 years, which indicates a growing interest of PRP in treatment for long-bone delayed union and nonunion. And we excluded those studies that patients in control group received surgery combined other treatment, including biologics and physiotherapy, to reduce bias of analysis. In our opinion, current literature is representative of the tendency for delayed union and nonunion.

As severe complications of a fracture, delayed union and nonunion could result in functional impairment, significant morbidity, and loss of quality of life for the afflicted patient (26). The causes of long-bone delayed union and nonunion were various (27). Delayed union and nonunion are two phases of bone healing disorders (28). The delayed union might result in nonunion or union (29). However, the treatment of delayed union is not clearly defined (30). Most doctors believe that interventions should be taken as soon as possible to treat delayed union to avoid the occurrence of nonunion (12). The types of nonunion include hypertrophic, atrophic and oligotrophic nonunion (31). According to the different causes of nonunion, different surgeries were performed (32). The principle of operation is to cure the local infection, remove all scar tissue from between the fracture fragments, correct deformity, immobilize of the fracture with internal or external fixation, and add biological stimulus (3).

In past years, biological stimulus mainly includes autologous bone grafting and allograft bone transplantation. Recently, many alternative methods of biological stimulus, such as bioactive factors and PRP, have been implemented. Bioactive factors, such as bone morphogenetic protein-2(BMP-2), have been confirmed by many studies to promote bone healing (33). As the only growth factor approved by the Food and Drug Administration (FDA), BMP-2 has been widely used worldwide as a bone graft substitute (34). However, with the increasing worldwide clinical administration of BMP-2, the side effect profile has emerged (35). The side effects of BMP-2 include ectopic bone formation, osteoclast-mediated bone resorption, inflammation associated complications, wound hematoma, infection, and inappropriate adipogenesis (36–41). In addition, as a kind of biological preparation obtained by concentrating peripheral blood, PRP has replaced the autologous whole blood injection method in treatment for many diseases (42, 43).

Many basic experimental studies have demonstrated the osteogenic properties of PRP in treatment for bone defects and nonunion for decade years (44, 45). In addition, the effectiveness of local administration of PRP in bone defects has been confirmed by many animal studies (46, 47). In the majority of experimental bone repair studies, PRP was used in conjunction with biomaterial scaffolds, such as collagen scaffolds (48), poly(lactic-co-glycolic acid) /calcium phosphate cement scaffolds (49), polycaprolactone-tricalcium phosphate scaffolds,β-tricalcium phosphate scaffolds (50). Although there are many basic experimental researches about PRP in treatment for delayed union and nonunion, the clinical studies are very few. In our study, the use of PRP mainly includes local injection or combined surgery during operation.

Healing rate is the most crucial determinant of the effectiveness of PRP in treatment for long-bone delayed union and nonunion. From above meta-analysis, the healing rate of the PRP group was higher than that of the control group, which indicated that PRP is an effective alternative treatment for delayed union and nonunion. Currently, there were no studies directly comparing the difference in treatment efficiency between PRP injection alone, and PRP combined with surgeries. Based on the all-enrolled studies in this systematic review, the healing rates of patients treated with PRP injection alone or during the operation were 94.19 and 78.69% respectively. In our opinion, to shorten the treatment period and increase the healing rate, local administration of PRP, combined with surgeries, is recommended for nonunion, while PRP injection alone is recommended for the delayed union.

Bony union time is another critical determinant of the effectiveness of PRP in treatment for long-bone delayed union and nonunion. The results of meta-analysis showed that the healing duration of the PRP group was shorter than the control group. The types of bone healing include clinical healing and radiological healing (3). In addition, the process of bone fracture healing consists of hematoma formation, soft callus formation, hard callus formation, and remodeling (51). In our opinion, the growth factors released by PRP could switch the process of bone healing rather than shorten the process of bone healing. However, the relationship between bone healing and growth factors released by PRP needs further confirmation by experimental studies.

Four enrolled studies reported that PRP is useful to relieve pain in patients with delayed union and nonunion. Many previous studies reported that PRP is effective to relieve pain in treatment for various diseases, such as external epicondylitis of the humerus, osteoarthritis, and plantar fasciitis (42, 52, 53). From the studies of Ghaffarpasand et al. (14) and De Vitis et al. (24) we could find, the PRP could play better role of relieving pain in the early stage after fixation surgery, especially the first 3 months. Over time, the pain score of both PRP and control group reduced in similar level. This trend was identical with the functional outcome De Vitis reported. Therefore, we can infer, PRP has the ability of relieving pain symptoms, by promoting local bone tissue repair at the early stage after surgery, and further promotes functional recovery.

The complication is the most crucial determinant of the safety of PRP in treatment for long-bone delayed union and nonunion. In a large group of patients, only three kinds of complications were described. Two enrolled studies described, respectively, small subcutaneous hematoma and subcutaneous swelling at the injection site after PRP injection, which resolved spontaneously at subsequent evaluations (21). In addition, Ghaffarpasand et al. (14) reported that there was no statistical difference in postoperative infection, although there was a tendency toward increased risk in the PRP group (14). In our opinion, increased infection risk may be associated with postoperative management, as the local infection is also a risk factor of delayed union or nonunion. In addition, more clinical trials with a larger size and longer follow-up are needed to show the trends in postoperative infection.

There were several limitations of the present systematic review. The main weakness of this study is the limited number of randomized controlled studies. Majority of the included studies are retrospective non-randomized comparative or non-comparative studies. Secondly, although many related studies have been included, the sample size of each trial is small. Although we tried to enroll as many patients as possible, the outcomes may also be underpowered, thus limiting the conclusions.

## Conclusions

Deriving from the multiple centrifugations of the own peripheral blood, PRP could successfully promote the healing rate and relieve the pain of patients with delayed union and nonunion. Although the limited reported studies, administration of PRP during operation or alone, is recommended as adjuvant therapy in treatment for long-bone delayed union and nonunion. However, the long-term adverse side and functional outcome in the administration of PRP still need further large-scale trials and long-term follow-up.

## Data Availability Statement

The original contributions presented in the study are included in the article/supplementary material, further inquiries can be directed to the corresponding author/s.

## Author Contributions

SL: conceptualization and writing—original draft preparation. FX and RL: methodology and formal analysis. RL: software. SL and FX: validation, resources, and data extraction. FX: investigation and writing—review and editing. ML: supervision. All authors have read and agreed to the published version of the manuscript.

## Conflict of Interest

The authors declare that the research was conducted in the absence of any commercial or financial relationships that could be construed as a potential conflict of interest.

## Publisher's Note

All claims expressed in this article are solely those of the authors and do not necessarily represent those of their affiliated organizations, or those of the publisher, the editors and the reviewers. Any product that may be evaluated in this article, or claim that may be made by its manufacturer, is not guaranteed or endorsed by the publisher.
